# Length of Stay in Emergency Department by ICD-10 Specific and Non-Specific Diagnoses: A Single-Centre Retrospective Study

**DOI:** 10.3390/jcm12144679

**Published:** 2023-07-14

**Authors:** Bartosz Miazgowski, Cezary Pakulski, Tomasz Miazgowski

**Affiliations:** 1 Doctoral School, Pomeranian Medical University in Szczecin, 71-252 Szczecin, Poland; 2 Department of Anesthesiology, Intensive Therapy and Emergency Medicine, Pomeranian Medical University in Szczecin, 71-252 Szczecin, Poland; 3 Department of Propaedeutic of Internal Diseases and Arterial Hypertension, Pomeranian Medical University in Szczecin, 71-252 Szczecin, Poland

**Keywords:** length of stay, emergency department, ICD-10 classification, non-specific diagnoses

## Abstract

The definition of non-specific presentation at a hospital emergency department (ED) has not yet been formally established. The purpose of this study was to assess the relationships between primary ED diagnoses identified by ICD-10 codes and ED length of stay (LOS). Over the course of three years, we examined 134,675 visits at a tertiary hospital. LOS was examined in groups with specific (internal, surgical, neurological, and traumatic diseases) and non-specific diagnoses. Our secondary objective was to measure LOS by age, day of the week, time of day, and season. The median LOS was 182 min (interquartile range: 99−264 min). LOS was 99 min in the traumatic group, while it was 132 min in the surgical group, 141 min in the non-specific group, 228 min in the internal medicine group, and 237 min in the neurological group. Other determinants of LOS were age, revisits, day of the week, and time of arrival—but not a season of the year. In the non-specific group (21% of all diagnoses), the percentage of hospitalizations was higher than in the specific groups. Our results suggest that in clinical practice, the non-specific group should be redefined to also encompass diagnoses from ICD-10 Chapter XXI (block Z00–Z99).

## 1. Introduction

Longer lengths of stay (LOS) and overcrowding in hospital emergency rooms (ED) have become serious issues for public healthcare systems around the world. The ED-LOS has been developed into a popular key performance indicator (KPI) that decision makers can use to systematically monitor and control ED performance in order to address this problem [1]. Long LOS have been found to pose a threat to patient safety [2], as they can lead to delayed care, unsatisfied patients, patients who leave before their treatment is finished, an increase in the likelihood of medical errors [3], and exhaustion in ED healthcare staff [4]. 

There are numerous causes of long-term ED-LOS that have been found; for instance, the majority of patients who attend emergency departments are treated and released without being admitted to the hospital, and a sizable portion of these instances involve non-urgent conditions—which may suggest problems with community access to primary, specialist, and preventive care [2]. Other factors—such as a lack of available hospital beds after leaving the ED [5], unintentional duplicate orders by physicians [6], and unnecessary blood chemistry testing and radiological imaging [7], as well as the patient’s age [8]—have also been associated with long LOS. Some reports suggest that LOS may be associated with admissions to ED during weekend days [9] and during late afternoon or night hours [8]. While several studies have evaluated LOS in relation to the initial ED diagnosis, only a few of these studies have assigned diagnoses to ICD-10 classification codes [10,11,12]—yielding ambiguous conclusions. Specifically, the LOS of patients with nonspecific diagnoses classified according to the ICD-10 remains unexplored.

Our study had two main objectives: Firstly, we aimed to evaluate the association between LOS and the most common primary ICD-10 diagnoses. To achieve this, we examined LOS in groups with specific and non-specific diagnoses. Our secondary objective was to measure LOS by age, day of the week, time of day, and season.

## 2. Materials and Methods

### 2.1. Study Design and Setting

This study is a retrospective, population-based cohort analysis of patients who were admitted to the ED at the Independent Public Teaching Hospital No. 1 (IPTH-1) of the Pomeranian Medical University in Szczecin, Poland, during January 2017–December 2019. IPTH-1 is a tertiary referral hospital consisting of 32 specialized clinical wards and about 800 beds that provides medical care across most medical specialties. At IPTH-1, all patient diagnoses are classified according to the International Classification of Diseases, Tenth Revision system. This study conforms to the Strengthening the Reporting of Observational Studies in Epidemiology (STROBE) guidelines [13].

### 2.2. Study Population

All adult arrivals to the ED at IPTH-1 (over the age of 18) were subjected to analysis. We used the resources of the hospital’s IT division to gather patient data. We did not use any exclusion criteria when analyzing the data. However, we removed any record elements that could be used to identify patients, such as names, social security numbers, addresses, and ID numbers, before exporting the data from the IT Department.

### 2.3. Methods

Our analysis focused on several key factors, such as age, time of admission and discharge, number of ED visits by day of the week, time of day, season, number of repeated visits, and hospital admission rates. The length of stay in the ED was calculated using the exact dates and times of admission and discharge as recorded in the hospital’s IT system. In total, there were 3859 primary diagnoses recorded between 2017 and 2019, classified according to the ICD-10 three- and four-character codes.

We then compiled a list of the most frequent diagnoses, which allowed us to distinguish four groups of patients with specific (disease) diagnoses typical for a given medical specialty—including those with internal, surgical, neurological, and traumatic diseases or injuries, as well as one group with non-specific (symptomatic) diagnoses. Patients in the non-specific group exhibited nonspecific signs, symptoms, and abnormal clinical and laboratory findings not elsewhere classified (Chapter XVIII, block R00-R99). We also included patients with selected diagnoses from ICD-10 Chapter XXI (block Z00-Z99; Factors influencing health status and contact with health services) in this group. For hospitalized patients, we verified the wards that had admitted the patients in the list of the most frequent diagnoses, and the degree of agreement between assignment to a specific disease group and admission to a dedicated target ward was 87.4%. In addition to these analyses, we also assessed LOS in relation to the day of the week, time of day, and season. The ED at IPTH-1 operates 24 h a day, with a full staff on weekdays from 8:00 a.m. to 3:45 p.m. and on-duty staff on weekdays from 3:45 p.m. to 8:00 a.m., as well as on weekends and holidays.

As the study did not involve the use of sensitive data from participating patients, the local Bioethics Committee approved the implementation without requiring formal opinions or written consent from the patients to participate.

### 2.4. Statistical Analysis

Descriptive statistics were presented as medians and interquartile ranges (IQR) or means and standard deviations for continuous variables and frequency distributions for categorical variables. The data were checked for normality using the Shapiro–Wilk test. Between groups, comparisons were made using a chi-square test or Fisher’s exact test for categorical variables and a Mann–Whitney U test for continuous variables. ED LOS between the years 2017, 2018, and 2019 was compared using the Kruskal–Wallis test.

Correlations between pairs of quantitative variables were analyzed using Spearman’s rho correlation. Multiple linear regressions were performed to model the relationship between the explanatory variables and the outcome variables. Statistical analyses were performed using IBM SPSS Statistics version 27 (SPSS Inc.; Chicago, IL, USA). Statistical significance was defined as *p*-value < 0.05.

## 3. Results

### 3.1. Baseline Characteristics

**Figure 1:**
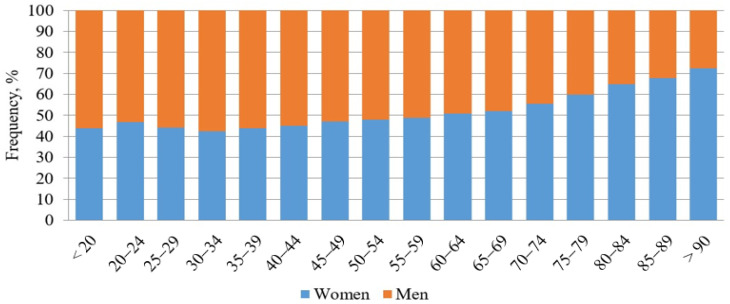
ED visits by age and sex.

As shown in Figure 1, ED visits were more common among men up to the age of 55, while they were more common among women over 70. From the beginning of 2017 to the end of 2019, there were 134,675 ED visits (122.9 ± 19 visits per day) including 67,573 women and 67,102 men. The number of visits in each year was 44,749 (33.33% of all visits in 3 years) in 2017, 45,697 (33.93%) in 2018, and 44,229 (32.84%) in 2019. There were no significant differences between the number of admitted women and men during the analyzed period. The total rate of repeated visits was 32%, with similar frequency in both sexes.

The frequency of ED visits varied depending on the day of the week, with most admissions occurring on Mondays and the least on Saturdays and Sundays. The mean number of ED visits on working days was over 28% higher than on non-working days (*p* < 0.001). Patients were most frequently admitted between 08:00 and 16:00, during the hours when the medical team was fully staffed. About 40% of patient arrivals were out of duty hours (16:00–08:00), of which 8% were at night (24:00–08:00). The number of ED visits in the winter (December to February) and autumn (September to November) months was significantly lower (*p* < 0.001) compared to the spring (March to May) and summer (June to August) months. The differences between arrival rates in autumn and winter as well as spring and summer were not significant.

### 3.2. Analysis of LOS

**Figure 2:**
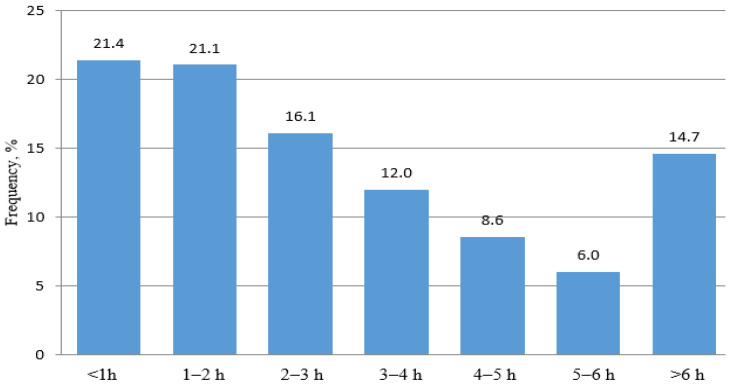
Frequency distribution of EDLOS in one-hour time intervals.

Throughout the study period, the median ED LOS was 182 min (IQR: from 99 to 264 min). Figure 2 shows the frequency distribution of LOS in one-hour time intervals from 2017 to 2019. The largest group of patients (42.5%) had LOS of less than 2 h. As the length of stay increased from 2 to 6 h, the number of patients decreased. Overall, 71% of patients had LOS under 4 h and 85% under 6 h.

Correlation analyses revealed that LOS was weakly but significantly associated with age (r = 0.201; *p* < 0.001). Multiple regression analyses showed that LOS > 4 h was associated with age (β = 2.04; *p* < 0.0001), ED visits during working days (β = 33.6; *p* < 0.0001), arrival during working hours (β = 12.1; *p* < 0.01), and revisits (β = 14.6; *p* < 0.005). However, this model, although significant (*p* < 0.001), only explained 9% of the variation in LOS.

### 3.3. LOS in the Specific and Non-Specific Groups

The causes for ED visits were next analysed in both the specific disease groups and the non-specific group, which together accounted for over 56% of all ED primary diagnoses (n = 3,859) from 2017 to 2019, as shown in Table 1. The largest number of cases were in the surgical and traumatic groups (24% each), followed by the non-specific (21%) and neurological and internal medicine groups (15% each). The most frequent diagnosis in the internal medicine group was I10 (Essential hypertension), in the surgical group—R10 (Acute abdomen), in the traumatic group—T92 (Sequelae of open wound of upper limb), in the neurological group—R42 (Dizziness and giddiness), and in the non-specific group—Z03.8 (Observation for other suspected diseases and conditions).

As shown in Table 2, ED LOS in the traumatic group was the shortest (160 min), while LOS in other groups was significantly longer: in the neurological group by 185 min (95% CI: 178–191; *p* < 0.001), in internal medicine by 158 min (95% CI: 151–164; *p* < 0.001), in the surgical group by 36 min (95% CI: 31–40; *p* < 0.001), and in the non-specific group by 24 min (95% CI: 19–28; *p* < 0.001). In 2019, compared to 2017, the mean LOS increased in most groups—particularly in the non-specific (by 28%) and surgical groups (by 16%). The percentage of hospitalizations was the highest in the non-specific group and lowest in the traumatic group.

Furthermore, we calculated LOS in each diagnostic subgroup for hospitalized and non-hospitalized patients (Table 3). Generally, in patients admitted to the hospital with specific diagnoses, LOS were shorter in comparison to patients leaving the ED without hospitalization. However, these differences were significant only in the surgical group (by 12.4 min; 95% CI: 2.96 to 21.8). A similar pattern was also observed in the non-specific group (LOS shorter in hospitalized patients by 9 min; 95% CI: 2.62 to 14.9; *p* = 0.02).

## 4. Discussion

To the best of our knowledge, this is the first study providing a comprehensive analysis of EDLOS in relation to ICD-10 primary diagnoses. In this report, we assessed LOS in five main clinical groups composed of the most frequent diagnoses—highly specific for each group. As the frequency of individual primary diagnoses may vary across EDs [14,15], the approach used here seems more appropriate for application in studies assessing LOS at the level of individual EDs with specific profiles (in terms of specific populations, availability of specialized hospital beds, number of medical staff, etc.)—especially when LOS is used as a KPI. In our study, the mean LOS was 3 h 2 min. Several countries, but so far not Poland, have implemented a 4 h [16,17,18] or a 6 h [19] rule as the target for 80–95% of patients. Applying these cutoffs to our cohort, LOS were suboptimal (71%) in the 4 h rule [17,18] and optimal (85%) in the 6 h rule [19].

The results of the current study clearly indicate that LOS largely depended on the cause of the ED visits. The longest LOS were observed in groups with neurological and internal diseases, and were the shortest in the traumatic group. Among the five groups of analysed diagnoses, the non-specific group seems to be of particular interest. It included patients who arrived to the ED due to nonspecific signs, symptoms, and abnormal clinical and laboratory findings (ICD-10, Chapter XVIII), as well as factors influencing health status and contact with health services (Chapter XXI). Overall, non-specific initial diagnoses were established in 21% of patients, with as many as 29% of these patients admitted to the hospital ward—while for comparison, the percentage of hospitalizations in the traumatic and surgical groups together was only 16%. Moreover, in the non-specific group, LOS were relatively shorter in comparison to other groups (except for traumatic cases)—especially in patients admitted to the hospital. These findings may suggest that in this group, ED physicians tend to make an earlier decision concerning hospitalization instead of performing further diagnostic procedures. It can therefore be assumed that at least some of these hospitalizations could have been caused by the need for longer observation and in-depth diagnostics in order to establish a more precise diagnosis, rather than the urgency of admission to the ward. In the study data, the percentage of hospitalizations was 22%—higher than the 10–14% reported in the United States [20], Portugal and Slovenia [21], or some Polish centres [22]. On the other hand, in Australia, 28% of all presentations to the ED in 2021–2022 ended in hospital admission, and this proportion varied across states and territories from 23% to 34% [23]. Even higher rates were reported in some European countries, including Denmark (46%) and Norway (69%) [21].

Generally, the problem of non-specific presentation in the ED still remains important and topical, and its definition has not yet been formally established [15]. Nonetheless, research into factors associated with non-specific ED diagnoses is scarce, even though they may encompass from 15% [24] to 37% [25] of all ED visits. These discrepancies may be due to differences in the methods used to identify such a group. For example, some studies use the clinical classification software (CCS) for ICD-10—a tool that groups diagnosis and ICD-9 procedure categorization schemes, in which all diagnoses that are classified as residual codes or symptoms, signs, ill-defined conditions, and factors influencing health status by the CCS are summarized as non-specific diagnoses, using such labels as “generally degraded health status” or “fever of unknown origin” [12,24].

We found that LOS were also related to the day of the week and the time of arrival to the ED. Most visits were registered on Mondays and other working days, and the least on Saturdays and Sundays. Overall, the average number of visits on working days was almost 30% higher than on non-working days. Most often, patients reported to the ED from 08:00 to 16:00, and least often at night. The LOS was significantly longer when visits took place on working days and during daylight hours, i.e., under conditions with full medical staff. There are divergent opinions in the literature on the impact of the day and time of visit on the length of stay in the emergency department; some authors report longer LOS at night compared to daytime visits [8,26]. In the study of Otto et al. [14], LOS were the longest on Mondays and the shortest on weekends, with the time of day being irrelevant. On the other hand, other reports have not shown any significant association between LOS and these factors [27,28]. 

We found no correlation between ED LOS and the season of the year, despite the fact that there were significantly more visits to the emergency department in the spring and summer months compared to other months. Similar results were also presented by Lee et al. [28]. Rather surprisingly, and so far alone, Yang et al. [26] found the longest LOS during the winter months—but only patients with a very urgent or urgent reason for admission were included in this analysis.

The current study has several limitations that should be considered when interpreting its results: Firstly, the findings are based on data from a single ED, and therefore may not be generalizable to other centres. Secondly, due to the lack of data in IT, the study did not analyse the time taken for laboratory and imaging tests, as well as specialist consultations, which are known to be significant determinants of LOS [2,27,28,29,30]. Due to the same reason, we were unable to evaluate LOS in relation to bed blocks, which are a common cause of long LOS [1,5,14,31]—particularly the waiting time for an available inpatient bed and the time of the disposition decision. Thirdly, the study analysed only the primary diagnoses established in the emergency department, and not the final diagnoses determined at discharge from the hospital. It has been estimated that there is a discrepancy of 15–30% between the primary and final diagnoses [3,15,24,30]. Therefore, some cases may have been classified into the wrong group or not included in any of them at all. This is particularly relevant for the non-specific group, where hospital observation and in-depth diagnostics would probably have provided a more precise diagnosis. On the other hand, the study’s strengths include its large sample size (almost 135,000 ED visits) and long duration (3 years), which allowed for the analysis of a diverse range of diagnoses across large clinical groups.

In conclusion, the study found that ED LOS was positively correlated with age and was significantly affected by the day of the week and time of arrival, with longer LOS observed during working days and daylight hours. The shortest LOS was found in patients with injuries and non-specific diagnoses, while the longest was found in the neurological group. The non-specific group had the highest percentage of hospitalizations. However, caution should be exercised in comparing these results with other studies, as the definition of the nonspecific group may differ across studies.

## Figures and Tables

**Table 1:** Most common ICD-10 diagnoses in specific and non-specific groups.

Internal Medicine		Surgical		Traumatic		Neurological		Non-Specific	
ICD-10	n	ICD-10	n	ICD-10	n	ICD-10	n	ICD-10	n
I10	2061	R10	3040	T92	2548	R51	1764	Z03.8	1395
R10.4	1287	S61	2330	T93	1955	R42	1074	Z76.9	1392
R19.8	979	S52	1760	S02	1747	R55	952	R42	1074
R07	866	S63	1533	S90	1546	G40.0	764	R51	1001
D38	575	S93	1292	S00	1523	I63	716	R10	946
J18	538	M54	1280	S60	1444	I64	608	R11	942
I48	486	M23	935	M70	1312	R29	528	R55	938
I50	268	S83	930	S01	1103	M47	512	R07	865
E05	189	M17	740	S40	420	G54	506	R53	820
J15	122	S80	669	S43	340	G44	486	R06.4	610
J45.9	108	S92	598	T00	308	G98	310	R04.0	509
I25	102	S66	521	S09	201	R26	309	Z76.8	235

I10, Essential (primary) hypertension; R10.4, Other and unspecified abdominal pain; R19.8, Other specified symptoms and signs involving the digestive system and abdomen; R07, Pain in throat and chest; D38, Neoplasm of uncertain or unknown behaviour of middle ear and respiratory and intrathoracic organs; J18, Pneumonia, organism unspecified; I48, Atrial fibrillation and flutter; I50, Heart failure; Thyrotoxicosis; J15, Bacterial pneumonia, not elsewhere classified; J45.9, Asthma, unspecified; I25, Atherosclerotic cardiovascular disease; R10, Abdominal and pelvic pain; S61, Open wound of finger(s) without damage to nail; S52, Fracture of forearm; S63, Dislocation, sprain and strain of joints and ligaments at wrist and hand level; S93, Dislocation, sprain and strain of joints and ligaments at ankle and foot level; M54, Dorsalgia; M23, Internal derangement of knee; S83, Dislocation, sprain and strain of joints and ligaments of knee; M17, Gonarthrosis; S80, Superficial injury of lower leg; S92, Fracture of foot, except ankle; S66, Injury of muscle and tendon at wrist and hand level; T92, Sequelae of injuries of upper limb; T93, Sequelae of injures of lower limb; S02, Fracture of skull and facial bones; S90, Superficial injury of ankle and foot; S00, Superficial injury of head; S60, Superficial injury of wrist and hand; M70, Soft tissue disorders related to use, overuse and pressure; S01, Open wound of head; S40, Superficial injury of shoulder and upper arm; S43, Dislocation, sprain and strain of joints and ligaments of shoulder girdle; T00, Superficial injuries involving multiple body regions; S09, Other and unspecified injuries of head; R51, Headache; R42, Dizziness and giddiness; R55, Syncope and collapse; G40.0, Localization-related (focal)(partial) idiopathic epilepsy and epileptic syndromes with seizures of localized onset; I63, Cerebral infarction; I64, Stroke, not specified as haemorrhage or infarction; R29, Other symptoms and signs involving the nervous and musculoskeletal systems; M47, Spondylosis; G54, Nerve root and plexus disorders; G44, Other headache syndromes; G98, Other disorders of nervous system, not elsewhere classified; R26, Abnormalities of gait and mobility; Z03.8, Observation for other suspected diseases and conditions; Z76.9, Person encountering health services in unspecified circumstances; R42, Vertigo; R51, Facial pain NOS; R10, Abdominal and pelvic pain; R11, Nausea and vomiting; R55, Fainting; R07, Pain in throat and chest; R53, Malaise and fatigue; R06.4, Hyperventilation; R04.0, Epistaxis; Z76.8; Persons encountering health services in other specified circumstances.

**Table 2:** LOS and hospitalizations in groups with specific and non-specific diagnoses.

Group	n	Age (Years)	F/M (%)	LOS, Mean ± SD (min)	LOS, Median (IQR) (min)	Hospitalizations (%)
Internal medicine	11,476	63.6 ± 25	58/42	318.5 ± 255	228 (103–309)	17.7
Surgical	18,510	58.8 ± 23	51/49	196.3 ± 187	132 (45–171)	8.8
Traumatic	18,310	41.7 ± 22	39/61	160.1 ± 164	99 (23–169)	7.1
Neurological	11,534	62.6 ± 26	48/52	345.0 ± 253	237 (65–336)	21.5
Non-specific	15,826	56.9 ± 26	52/48	185.4 ± 191	141 (70–217)	29.3
All	134,675	52.4 ± 23	50/50	254.0 ± 205	212 (93–251)	22.0

For all comparisons between groups *p* < 0.001.

**Table 3:** LOS for each diagnostic subgroup for hospitalized and non-hospitalized patients.

Group	Hospitalization		No Hospitalization		*p*
	n	LOS (min)	n	LOS (min)	
Internal medicine	2031	311.5 ± 229	9445	321.4 ± 262	0.111
Surgical	1629	189.2 ± 169	16,881	201.6 ± 187	0.010
Traumatic	1300	158.2 ± 140	17,010	166.1 ± 159	0.082
Neurological	2480	343.3 ± 243	9054	349.0 ± 257	0.321
Non-specific	4637	180.4 ± 179	11,189	189.2 ± 181	0.024

## Data Availability

The data presented in this study are available on request from the corresponding author.
